# Ensemble disease gene prediction by clinical sample-based networks

**DOI:** 10.1186/s12859-020-3346-8

**Published:** 2020-03-11

**Authors:** Ping Luo, Li-Ping Tian, Bolin Chen, Qianghua Xiao, Fang-Xiang Wu

**Affiliations:** 1 Division of Biomedical Engineering, University of Saskatchewan, Saskatoon, S7N 5A9 Canada; 2 School of Information, Beijing Wuzi University, Beijing, 101149 China; 3 School of Computer Science, Northwestern Polytechnical University, Xi’an, 710072 China; 4 School of Mathematics and Physics, University of South China, HengYang, 421001 China; 5 Department of Computer Science, University of Saskatchewan, Saskatoon, S7N 5C9 Canada; 6 School of Mathematics and Statistics, Hainan Normal University, Haikou, 571158 China; 7 Department of Mechanical Engineering, University of Saskatchewan, Saskatoon, S7N 5A9 Canada

**Keywords:** Disease gene prediction, Sample-based networks, Ensemble learning, Network centrality, Protein-protein interaction network

## Abstract

**Background:**

Disease gene prediction is a critical and challenging task. Many computational methods have been developed to predict disease genes, which can reduce the money and time used in the experimental validation. Since proteins (products of genes) usually work together to achieve a specific function, biomolecular networks, such as the protein-protein interaction (PPI) network and gene co-expression networks, are widely used to predict disease genes by analyzing the relationships between known disease genes and other genes in the networks. However, existing methods commonly use a universal static PPI network, which ignore the fact that PPIs are dynamic, and PPIs in various patients should also be different.

**Results:**

To address these issues, we develop an ensemble algorithm to predict disease genes from clinical sample-based networks (EdgCSN). The algorithm first constructs single sample-based networks for each case sample of the disease under study. Then, these single sample-based networks are merged to several fused networks based on the clustering results of the samples. After that, logistic models are trained with centrality features extracted from the fused networks, and an ensemble strategy is used to predict the finial probability of each gene being disease-associated. EdgCSN is evaluated on breast cancer (BC), thyroid cancer (TC) and Alzheimer’s disease (AD) and obtains AUC values of 0.970, 0.971 and 0.966, respectively, which are much better than the competing algorithms. Subsequent de novo validations also demonstrate the ability of EdgCSN in predicting new disease genes.

**Conclusions:**

In this study, we propose EdgCSN, which is an ensemble learning algorithm for predicting disease genes with models trained by centrality features extracted from clinical sample-based networks. Results of the leave-one-out cross validation show that our EdgCSN performs much better than the competing algorithms in predicting BC-associated, TC-associated and AD-associated genes. de novo validations also show that EdgCSN is valuable for identifying new disease genes.

## Background

Disease gene prediction is a critical yet challenging task. It helps us understand the mechanisms of diseases, find therapeutic targets, and develop novel treatment strategies [1]. During the past decades, disease gene prediction has gained great development. Many computational algorithms have been developed to predict disease genes so that the cost and time for in-depth validation could be maximumly reduced.

Among the various types of data that have been used to predict disease genes, protein-protein interactions (PPIs) are the most widely used evidence. On the one hand, interacting proteins (genes) usually have similar functions, which means algorithms can predict new disease genes based on their relationships with known disease genes in the PPI network. On the other hand, due to the network property of PPIs, most network analysis algorithms can be used to predict disease genes from PPI networks. For example, earlier methods, such as RWR, performed the random walk on PPI networks to predict disease genes [2]. Gillis et al. used degree centralities to rank all the genes [3].

However, PPIs are dynamic during the life time of cells, and not all PPIs exist in all the tissues. Static PPI networks downloaded from online databases contain lots of false positives which limit the performance of the methods that directly use them [4]. Thus, many studies integrate static PPI networks with disease-related data, such as GWAS and gene expression data, to improve the prediction accuracy [5–7]. This leads to two types of approaches. The first type of approaches weights PPI networks with disease-related data, and predicts candidate genes from the weighted networks. For instance, Wang et al. searched dense modules from a PPI network weighted by gene expression and GWAS data [6]. Our previous study trained a regression model with features extracted from a PPI network weighted by differential co-expression [8]. The second type of approaches constructs heterogeneous networks and combines them with PPI networks to enhance the prediction. For example, Chen et al. combined gene co-expression networks and pathway coexist networks with PPI networks to predict disease genes [9, 10]. Singh-Blom et al. trained a biased SVM with features extracted from phenotype-phenotype networks and PPI networks [11] to predict disease genes. Despite their success, the discussed approaches still use PPI networks with false positive interactions, which contain inaccurate topological structures. PPI networks downloaded from different databases might affect the prediction results.

To solve these issues, in our previous study, gene expression data of clinical samples have been used to construct sample-specific PPI networks [12]. Each single sample-based network only contains the significant PPIs associated with the disease under consideration, which reduces the false positive interactions. A network that fuses all the single sample-based networks was used to predict the disease-associated genes, so that disease genes that function in different patients could all be identified. In this study, to further extend our research, an ensemble algorithm that predicts disease genes from clinical sample-based networks (EdgCSN) is proposed. Meanwhile, Katz centrality is used instead of edge clustering coefficient to better extract local structural information from the sample-based networks.

## Methods

**Fig. 1:**
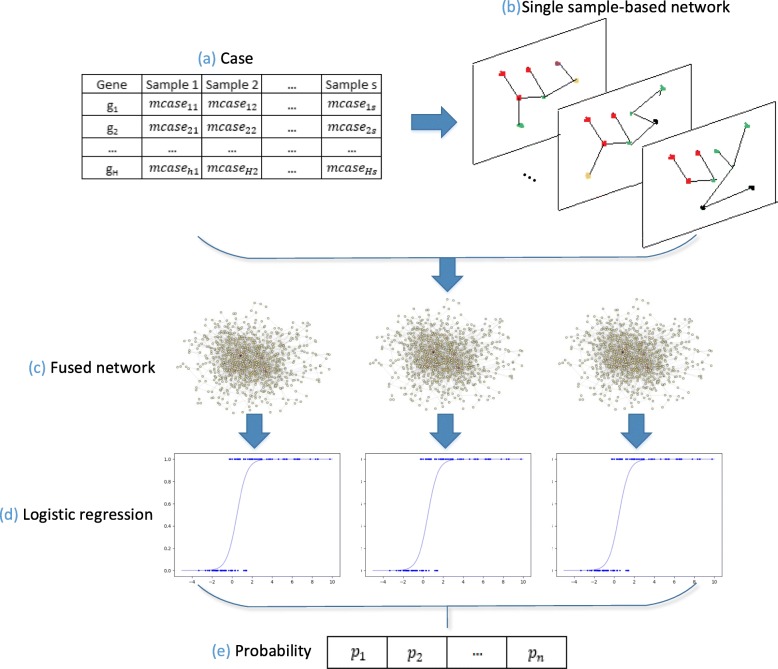
Work flow of the algorithm. (**a**) Obtain gene expression data of case samples; (**b**) Construct single sample-based networks; (**c**) Fuse sample-based networks based on the clustering results; (**d**) Perform prediction on each fused network; (**e**) Combine the prediction results in (d) to generate the final prediction

Figure 1 depicts the work flow of EdgCSN which is explained as follows. (a)-(b). A single sample-based network is constructed for each case sample by combining clinical samples and the universal static PPI network. (c). The case samples are clustered into a few groups and single sample-based networks of the samples in the same group are fused to one network. (d). A logistic model is trained by the centrality features extracted from each fused network, and the probability of each gene being disease-associated is predicted. (e). The maximum probability of a gene calculated from all the logistic models is regarded as its probability of being disease-associated. In the following subsections, details of the five steps in Fig. 1 are first discussed. Then, the data sources and evaluation metrics are explained.

### Sample-based networks

To obtain the most informative PPIs and remove the false positive ones, sample-based networks are used in this study instead of the universal static PPI networks. In addition, since the real caustic genes of different patients may not be the same, case samples are divided into different clusters so that patients with distinct conditions are analyzed separately. Specifically, three steps are performed to obtain the sample-based networks.

1. A single sample-based network is constructed for each case sample;
2. Case samples are classified into different clusters;
3. Networks of the samples in the same cluster are fused together.

For the first step, we assume that a PPI exists in a single sample-based network *N*_*s*_ only if the two interacted proteins are both activated in sample *s*. Concretely, a gene *i* in a case sample *s* is considered being activated if

1 $$ \text{mcase}[i,s] \ge \lambda * mean(\text{mcntl}[i])  $$

where mcase[*i*,*s*] is the expression value of gene *i* in sample *s*, and *mean*(mcntl[*i*]) is the mean expression value of gene *i* over all control samples. To construct *N*_*s*_, every edge (*i*,*j*) in the static PPI network is validated and only the one with both *i* and *j* being activated is added to *N*_*s*_. Then, *S* single sample-based networks are constructed for the *S* case samples.

For the second step, hierarchical clustering is used to classify case samples into different clusters. Given two samples *s*_1_ and *s*_2_, their pairwise distance is calculated by

2 $$  dist(s_{1},s_{2}) = 1-\frac{(\mathbf{s}_{1}-\bar{\mathbf{s}}_{1})\cdot (\mathbf{s}_{2}-\bar{\mathbf{s}}_{2})}{\Vert \mathbf{s}_{1}-\bar{\mathbf{s}}_{1} \Vert_{2} \Vert \mathbf{s}_{2}-\bar{\mathbf{s}}_{2} \Vert_{2}}  $$

where **s**_1_ (**s**_2_) is a vector of expression values of genes in sample *s*_1_ (*s*_2_), and $\bar {\mathbf {s}}_{1}$ ($\bar {\mathbf {s}}_{2}$) is the corresponding average expression value. During the bottom-up process, distance between two newly formed clusters *u* and *v* is computed as follows

3 $$ Distance(u,v) = \max_{p \in u, q \in v} (dist(p,q))  $$

which is the maximum distance between samples in *u* and *v*. Let *dmax* denote the maximum distance among clusters, 0.7∗*dmax* is used as the threshold to select clusters from the resulted dendrogram.

For the third step, assuming all the *S* samples are classified into *l* clusters and the *t*-th cluster contains *S*_*t*_ samples, we have $S=\sum _{t=1}^{l}S_{t}$. The objective is to fuse the networks of the samples in the same cluster into one network. Although many network fusion methods have been published [13], most of them cannot efficiently fuse complex PPI networks, especially when the number of networks to be fused is more than 1,000. Thus, we propose a simple strategy which uses a threshold *ε* to determine whether an edge exists in the fused networks. An edge (*i*,*j*) is considered as significant only if it appears in at least *ε* single sample-based networks. Precisely, given a cluster with *S*_*t*_ samples, let *f*_*ij*_ be the number of times edge (*i*,*j*) appears in the *S*_*t*_ single sample-based networks. When *f*_*ij*_<*ε*, (*i*,*j*) is not included in the fused network, and when *f*_*ij*_≥*ε*, (*i*,*j*) is in the fused network. Finally, *l* fused networks are obtained for the *l* clusters, respectively.

### Model design

Given a biomolecular network, if disease genes are labeled as 1 and non-disease genes are labeled as 0, the disease gene prediction problem can then be formulated as a network labeling problem [14]. Let **x**=(*x*_1_,*x*_2_,…,*x*_*H*_) denote a set of binary labels of all the *H* genes in the biomolecular network. **x** is known as the configuration of the network, and the set *X* of all possible configurations is a random field. Based on our previous studies [8, 10, 15], a generalized model was proposed in [12] which predicted the probability of a gene *i* being labeled as 1 by

4 $$  P(x_{i}=1|x_{[-i]},\theta) = \frac{\exp(\theta \phi_{i})}{1+\exp(\theta \phi_{i})}  $$

where *θ* is a parameter vector and *ϕ*_*i*_ is the feature vector of gene *i* extracted from the biomolecular network labeled by a prior configuration **x**.

In [12], *ϕ*_*i*_ is a 7-dimensional feature vector which consists of a dummy feature (1) and three pairs of 0–1 centrality features: 0–1 degree centrality, 0–1 closeness centrality and 0–1 edge clustering coefficient. These three 0–1 centrality indices have shown their ability in characterizing discriminative features for classifying disease and non-disease genes. However, edge clustering coefficient can only capture the structural information between genes and their direct neighbors, and the relations between genes and their k-th order (*k*≥2) neighbors cannot be obtained. Since proteins usually form a complex or functional module to achieve a specific function [4], the k-th order neighbors should also be considered when the local structural information is extracted. Previous study also showed that the indirect neighbors were useful for disease gene prediction [16]. Thus, we replace edge clustering coefficient by Katz centrality in this study to leverage the local structure information between nodes and their higher order neighbors.

Given a labeled network *N*=(*V*,*E*), *V* is the set of nodes and *E* is the set of edges, the 0–1 degree centrality denoted by $C^{d}_{i0}$ and $C^{d}_{i1}$ are defined as follows

5 $$ C^{d}_{i0} = \sum_{(i,j) \in E} (1-x_{j}), \quad C^{d}_{i1} = \sum_{(i,j) \in E} x_{j}  $$

The 0–1 closeness centrality denoted by $C^{c}_{i0}$ and $C^{c}_{i1}$ are defined as

6 $$ \begin{aligned} C^{c}_{i0} &= \frac{1}{n_{0}-1} \sum_{j \in V, j \ne i}\frac{1}{dsp(i,j)} (1-x_{j}), \\ C^{c}_{i1} &= \frac{1}{n_{1}-1} \sum_{j \in V, j \ne i}\frac{1}{dsp(i,j)} x_{j} \end{aligned}  $$

where *dsp*(*i*,*j*) is the length of the shortest path between node *i* and *j*, *n*_0_ and *n*_1_ are the number of nodes labeled as 0 and 1, respectively

Katz centrality measures the relative influence of a node in the network [17]. It is defined by

7 $$ C^{k}_{i} = \sum_{k=0}^{\infty} \sum_{j=1}^{n} \alpha^{k} \left(A^{k}\right)_{ji}  $$

where *A* is the adjacency matrix of the network, *k* is the length of the path between *i* and *j*, *α* is a damping factor penalizes the impact node *j* on *i*. The longer the path, the smaller the impact node *j* is on *i*.

When *α* is properly chosen, Eq. (7) will converge as *k*→*∞*. However, when Katz centrality is used in this study, we care more about the information conveyed by paths with short distance (less than 5). Study in link prediction also showed that *k*=3 or *k*=4 can yield satisfactory performance [18]. Thus, *α* and *k* are chosen by grid search without the proof of convergence.

In previous studies, Katz centrality calculated from heterogeneous networks had been used to prioritize disease genes [11]. However, results of directly using Katz centrality were not better than existing methods, such as RWR [2]. To make Katz centrality suitable for disease gene prediction, we define the 0–1 Katz centrality as follows:

8 $$ \begin{aligned} C^{k}_{i0} &= \sum_{k=0}^{\infty} \sum_{j=1}^{n} \alpha^{k} \left(A^{k}\right)_{ji} (1-x_{j}),\\ C^{k}_{i1} &= \sum_{k=0}^{\infty} \sum_{j=1}^{n} \alpha^{k} \left(A^{k}\right)_{ji} x_{j} \end{aligned}  $$

Similar to 0–1 degree and 0–1 closeness centrality, the 0–1 Katz centrality measures the importance of a gene among disease genes and non-disease genes, respectively, which is more appropriate for disease gene prediction. The new feature vector of each gene is then defined as

9 $$ \phi_{i} = \left(1,C^{d}_{i0},C^{d}_{i1},C^{c}_{i0},C^{c}_{i1},C^{k}_{i0},C^{k}_{i1}\right)  $$

### Network labeling and benchmark selection

As discussed in the previous section, biomolecular networks are needed to be labeled by a prior configuration so that disease genes can be predicted. In this study, we use the *l* fused networks to predict disease genes, which means the known disease genes in these networks are labeled as 1 while other genes are labeled as 0. Then, the feature vectors of all genes can be extracted by Eq. (9).

In addition, to train the logistic models used for prediction, we also need a set of non-disease genes, which are used as negative instances. Unfortunately, no databases contain non-disease genes. Therefore, our previous strategy proposed in [19] is used to select the non-disease genes used in the training.

In [19], a disease gene network (DGN) was constructed with the disease-gene association data downloaded from OMIM [20]. In the DGN, each node is either a disease or a disease-associated gene. Diseases are connected with their associated genes, and two diseases are connected if they share one or more associated genes. Thus, diseases that are close to each other in the DGN have more chances to share similar disease genes, which means they are more likely to have similar mechanisms. If the length of the shortest path between two diseases is larger than a threshold *η*, they might not have similar mechanisms, and the disease genes of one disease could be regarded as non-disease genes of the other disease. With this strategy, a group of non-disease genes are obtained for the disease under study, and only non-disease genes that exist in all the *l* fused networks are selected. *η*=5 is chosen based on our previous experience.

Assuming *m* disease genes are known to be associated with the disease under study, we randomly select *m* genes from the set of non-disease genes, and these 2*m* genes form a set of gold standard genes. This process is performed 50 times and finally we obtain 50 sets of gold standard genes and regarded them as benchmarks.

### Ensemble prediction

Given *m* disease genes and *m* non-disease genes, features of these genes extracted from the *l* fused networks are used to train *l* logistic models, respectively. Equation (4) is then used to predict the probability of each gene being disease-associated in each fused network.

For each gene, $l^{'} (1 \le l^{'} \le l)\phantom {\dot {i}\!}$ probabilities are calculated. Considering that the caustic genes of different samples might be different, the obtained probabilities only reveal the potential of the gene being disease-associated in the corresponding clusters. Thus, for each gene, the ensemble strategy chooses the maximum value of the $\phantom {\dot {i}\!}l^{'}$ probabilities as its probability of being disease-associated.

### Datasets

In this study, datasets of breast cancer (BC), thyroid cancer (TC) and Alzheimer’s disease (AD) are used to evaluate the algorithm. The BC-associated genes and TC-associated genes are obtained from the Cancer Gene Census category (http://cancer.sanger.ac.uk/census) [21]. In total, 35 BC-associated genes and 33 TC-associated genes are used as the benchmarks. The AD-associated genes are obtained from MalaCards: The human disease database (http://www.malacards.org/). The database contains 182 potential AD associated genes ranked by their probability of being AD-associated in descending order. 39 of the first 50 genes exist in the static PPI network are used as benchmarks.

The gene expression data of BC and TC are downloaded from NCI Genomic Data Commons (GDC) [22], which measures the data by RNA-Seq. We download the data normalized by FPKM (Fragments Per Kilobase Million) and transform them to TPM (Transcripts Per Kilobase Million) by the strategy proposed in [23]. The expression data of Alzheimer’s disease (AD) are downloaded from Gene Expression Omnibus (GSE53697) [24], which are also measured by RNA-seq. The data normalized by RPKM (Reads Per Kilobase Million) are downloaded and transformed to TPM with the same strategy used for the data downloaded from GDC. TPM is chosen because it facilitates the comparison of the proportion of reads that are mapped to a gene in each sample and is usually better than FPKM and RPKM in cross-sample comparison, which helps us properly cluster all the samples. In total, the dataset of BC contains 1102 case samples and 113 control samples; the dataset of TC contains 502 case samples and 58 control samples; the dataset of AD contains 9 case samples and 8 control samples.

After downloading the gene expression data, four steps are performed to control the genes used in our study. (1). TPM values less than 1 are replaced by 0 because of the unreliability. (2). log2(TPM+1) is used instead of the original TPM values. (3). Genes expressed in less than 10% of samples (case and control) are removed. (4). Genes not existing in the PPI network are removed. In total, 14436 genes, 13959 genes and 13370 genes are left for BC dataset, TC dataset and AD dataset, respectively.

The static PPI network is downloaded from the InWeb_InB-ioMap database (version 2016_09_12) [25]. The database consists of more than 600,000 protein interactions collected from eight source databases, which insures that valuable protein interactions are not missed during the construction of the sample-based PPI networks. In this study, the proteins in the PPI network are mapped to their corresponding genes to form a gene-gene interaction network. In the paper, the term “PPI network” is used to represent the gene-gene interaction network because of simplicity.

### Evaluation metrics

In this study, a disease gene is regarded as positive while a non-disease gene is regarded as negative. Given a threshold *Γ*, a gene *i* with a probability *p*_*i*_≥*Γ* is predicted as positive, and otherwise it is predicted as negative. For all genes in the benchmark, the true positives (TP), false positives (FP), true negatives (TN), and false negatives (FN) are defined as follows

1. *TP*: a disease gene is predicted as a disease gene
2. *FP*: a non-disease gene is predicted as a disease gene
3. *TN*: a non-disease gene is predicted as a non-disease gene
4. *FN*: a disease gene is predicted as a non-disease gene

Then, we can calculate the true positive rate (TPR) and the false positive rate (FPR) of the prediction results by the following equations

10 $$ TPR=\frac{TP}{TP+FN}, \ FPR=\frac{FP}{TN+FP}  $$

To evaluate the algorithm, the receiver operating characteristic (ROC) curve is created by plotting the TPR against FPR with various *Γ*. The area under the ROC curve (AUC) is also used to evaluate the overall performance of the algorithm.

Since the number of genes used as benchmark is small, leave-one-out cross validation (LOOCV) is performed to calculate the probabilities of genes in the benchmark being disease-associated. With the 50 sets of gold standard genes, LOOCV is performed 50 times. In each round, the probabilities of the 2*m* genes being disease-associated are calculated, as well as the AUC value. The average AUC value is then used to evaluate the algorithm.

In addition, de novo validation is performed by ranking all the unknown genes in descending order by their average probabilities calculated by the models trained with the 50 sets of gold standard genes. The top 10 unknown genes are analyzed from published literature to illustrate the ability of EdgCSN in predicting new disease genes.

## Results

### Clustering

**Fig. 2:**
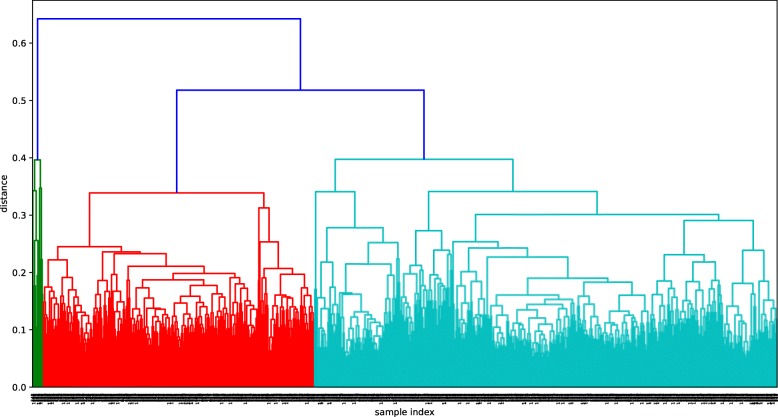
Hierarchical clustering dendrogram for BC

**Fig. 3:**
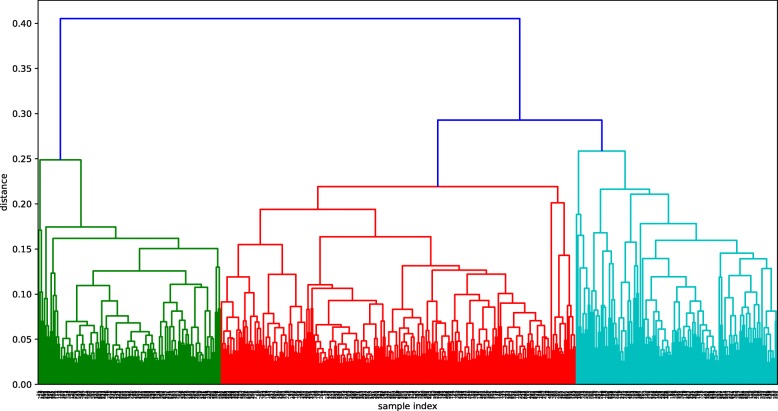
Hierarchical clustering dendrogram for TC

**Fig. 4:**
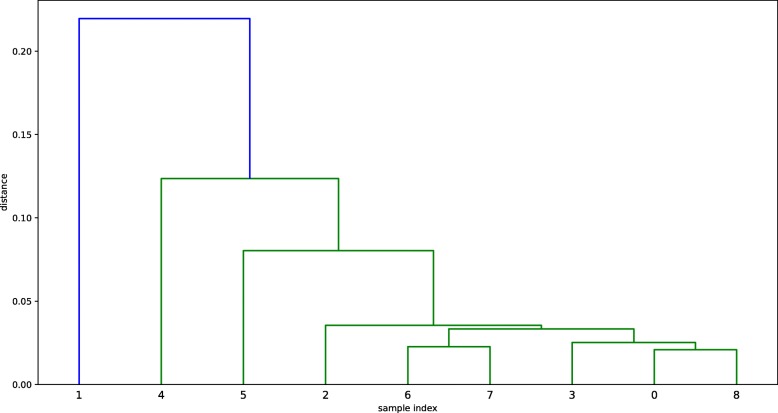
Hierarchical clustering dendrogram for AD

Figures 2, 3 and 4 show the dendrograms of the hierarchical clustering. BC and TC samples are divided to three clusters and AD samples are divided to two clusters. Thus, three fused networks are constructed for BC and TC, respectively, and two fused networks are constructed for AD.

### Sensitivity analysis

The performance of our algorithm is affected by four hyperparameters: *λ*, *ε*, *α* and *k*. The first two control the resulted fused networks. Based on our previous study, edges that exist in more than three networks were significant [12]. Thus, *ε*=3 is empirically chosen in this study. As for *λ*, since the RNA-seq data are normalized by TPM rather than DESeq2 [26], *λ* is searched from a new set {1.0,1.1,1.2,1.3,1.5}, which is different from the one obtained in our previous study. The other two hyperparameters control the information extracted by Katz centrality. To obtain the appropriate hyperparameters, *α* is searched from {0.1,0.2}, and *k* is searched from {1,2,3,4}, respectively.

Tables 1, 2 and 3 show the results of the grid search for BC, TC and AD, respectively. EdgCSN performs best for BC when *λ*=1.1,*α*=0.2,*k*=2 with an AUC=0.970; for TC when *λ*=1.11,*α*=0.1,*k*=2 with an AUC=0.971; for AD when *λ*=1.0,*α*=0.2,*k*=2 with an AUC=0.966. ‘-’ denotes that more than 10% known disease genes are not contained in the fused networks constructed by the corresponding hyperparameters.

**Table 1:** Sensitivity analysis

		k			
*λ*	*α*	1	2	3	4
1.0	0.1	0.867	0.961	0.873	0.878
1.0	0.2	0.869	0.966	0.889	0.870
1.1	0.1	0.883	0.967	0.890	0.903
1.1	0.2	0.881	**0.970**	0.909	0.896
1.2	0.1	0.845	0.957	0.877	0.898
1.2	0.2	0.846	0.958	0.892	0.894
1.3	0.1	0.787	0.938	0.819	0.842
1.3	0.2	0.787	0.940	0.841	0.842
1.5	0.1	0.777	0.938	0.813	0.775
1.5	0.2	0.777	0.938	0.786	0.816

The resulted AUC values obtained with different combinations of hyperparameters for BC

The highest AUC value is marked in boldface

**Table 2:** Sensitivity analysis

		k			
*λ*	*α*	1	2	3	4
1.0	0.1	0.716	0.966	0.839	0.790
1.0	0.2	0.713	0.967	0.795	0.802
1.1	0.1	0.729	**0.971**	0.800	0.746
1.1	0.2	0.728	0.969	0.744	0.779
1.2	0.1	0.809	0.954	0.748	0.776
1.2	0.2	0.808	0.953	0.652	0.792
1.3	0.1	0.621	0.962	0.779	0.786
1.3	0.2	0.620	0.960	0.662	0.794
1.5	0.1	0.412	0.965	0.809	0.720
1.5	0.2	0.411	0.963	0.645	0.679

The resulted AUC values obtained with different combinations of hyperparameters for TC

The highest AUC value is marked in boldface

**Table 3:** Sensitivity analysis

		k			
*λ*	*α*	1	2	3	4
1.0	0.1	0.808	0.964	0.809	0.763
1.0	0.2	0.809	**0.966**	0.764	0.705
1.1	0.1	0.665	0.956	0.757	0.685
1.1	0.2	0.665	0.957	0.596	0.636
1.2	0.1	0.564	0.938	0.809	0.605
1.2	0.2	0.563	0.939	0.608	0.596
1.3	0.1	0.508	0.914	0.810	0.674
1.3	0.2	0.508	0.914	0.608	0.614

The resulted AUC values obtained with different combinations of hyperparameters for AD

The highest AUC value is marked in boldface

All the three experiments obtain their best AUC values when *k*=2, and a smaller or higher *k* would significantly affect the performance of the algorithm. These results indicate that local structural information contained within the second order neighborhood is valuable for disease gene prediction. Other disease gene prediction algorithms that use topological structure of biomolecular networks could also further include these information to improve their prediction.

### Comparison

EdgCSN is compared with three algorithms: the Re-balanced algorithm of Chen et al. [10], the AIDG algorithm of Tang et al. [27], and our previous algorithm dgCSN [12]. Re-balanced method combined multiple types of biomolecular networks to predict cancer-related genes, and AIDG used sub-cellular localization to purify universal PPI networks. These algorithms have been shown better than many classical methods, such as the RWR method [2], the DIR method [28] and the ToppNet [29].

**Fig. 5:**
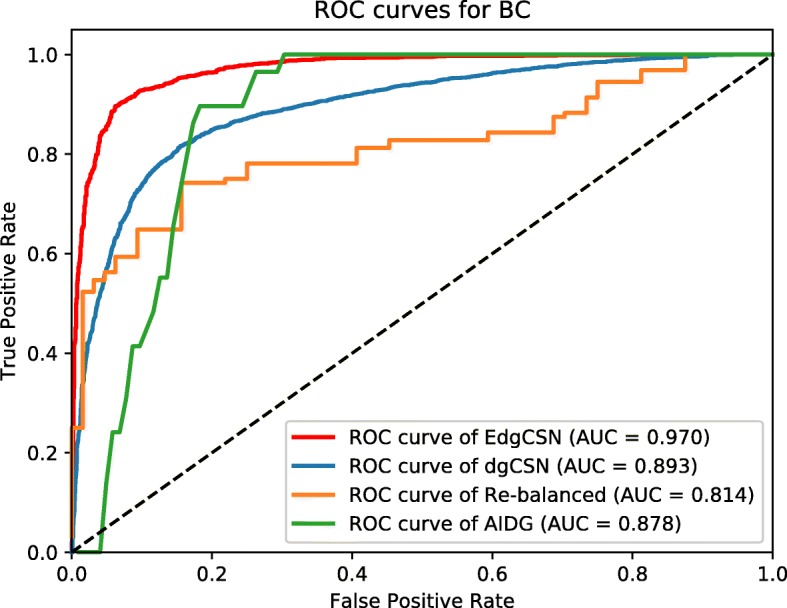
ROC curves for BC

**Fig. 6:**
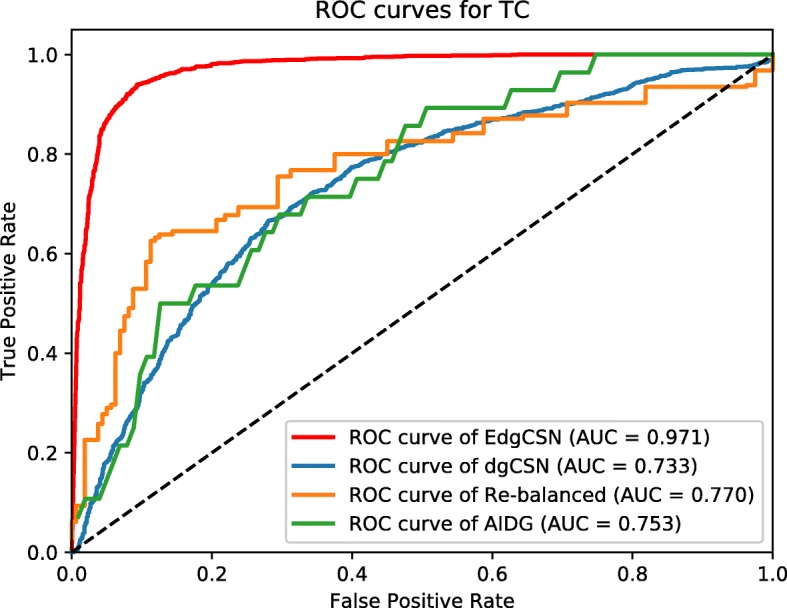
ROC curves for TC

**Fig. 7:**
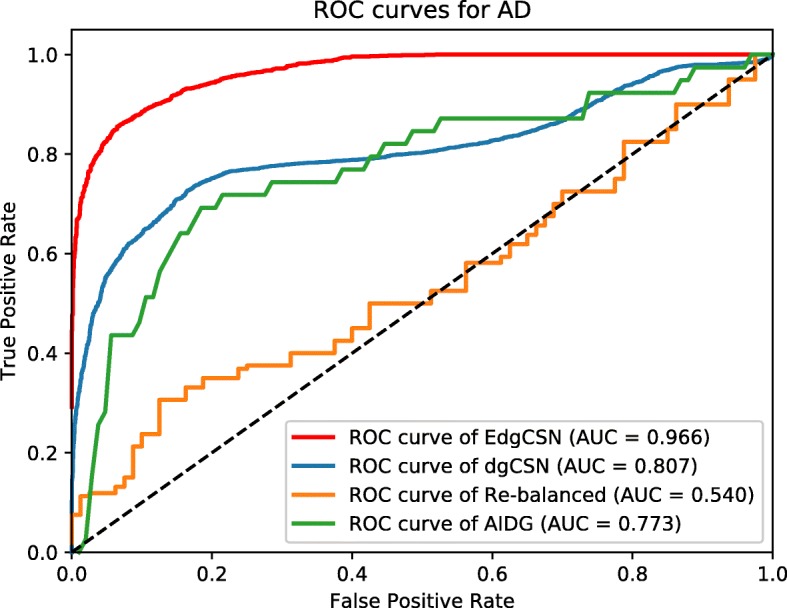
ROC curves for AD

The resulted ROC curves for BC, TC, and AD are depicted in Figs. 5, 6, 7, respectively. The AUC values of EdgCSN for BC, TC and AD are 0.970, 0.971 and 0.966, respectively, which are much better than those of the competing algorithms. For BC, our EdgCSN is 7% more accurate than the competing algorithms, and for TC and AD, EdgCSN is 20% more accurate than the other three algorithms.

### de novo validation

To validate the performance of EdgCSN in predicting new disease genes, unknown genes are ranked in descending order by their average probabilities of being disease-associated predicted by the 50 sets of genes in the benchmark. The top 10 predictions are further searched in existing literature to find out if they are associated with the disease under study.

Table 4 shows the top 10 predictions of the three diseases. Functions of the genes that have not been studied in existing literature are left blank. Most of the genes have been analyzed as disease-associated in existing studies, especially for BC, where all the 10 genes have been studied in the existing literature. For TC, although only 5 of the 10 genes have been studied, 3 of the 5 genes that have not been studied (‘CEP72’, ‘CEP131’ and ‘GPR83’) belong to the Centrosomal Protein family and G Proteincoupled Receptor respectively. Many proteins belong to these families are closely related to cancers [30], which means ‘CEP72’, ‘CEP131’ and ‘GPR83’ might be predicted as being TC-associated in the future.

**Table 4:** Top 10 unknown genes

Gene Name	Function	Reference
**BC**		
CREBBP	Potential disease gene	[31]
NBN	Potential disease gene	[32]
PARP1	Potential biomarker	[33, 34]
NCOR2	Potential biomarker	[35]
RXRA	Potential therapeutic target	[36]
WRN	Potential disease gene	[37]
EXO1	Potential disease gene	[38]
NCOA3	Potential disease gene	[39]
RMI2	Potential disease gene	[40]
TOPBP1	Potential therapeutic target	[41]
**TC**		
HRAS	Potential disease gene	[42]
HAUS7		
CEP72		
GTF2I	Potential disease gene	[43]
BCLAF1	Potential disease gene	[44]
HAUS3		
FGFR1OP	Potential disease gene	[45, 46]
CEP131		
GPR83		
ALMS1	Potential disease gene	[47]
**AD**		
MAP2	Potential disease gene	[48]
DPYSL3		
ERRFI1	Potential disease gene	[49]
DAB2	Potential disease gene	[50]
AMPH	Potential disease gene	[51]
SYN1	Potential disease gene	[52]
SYT9	Potential disease gene	[53]
AXIN1		
PRNP	Potential disease gene	[54]
AAK1	Potential disease gene	[55]

## Discussion

Many algorithms have been proposed to predict disease genes, and most of them rely on PPI networks to achieve the prediction. However, PPI is dynamic and tissuespecific, static PPI networks downloaded from online databases contain many false positives, and directly using them would limit the accuracy of disease gene prediction. Moreover, for patients with a specific disease, their disease states might be driven by different subset of disease genes, and analyzing their data together might affect the identification of rarely mutated disease genes.

Therefore, in this study, an ensemble algorithm is proposed to predict disease genes from clinical sample-based networks. The algorithm first constructs single sample-based networks by combining clinical samples and a universal static PPI network. A group of networks which contain disease-related PPIs are generated. Then, case samples are divided into different clusters and networks belong to the samples in the same cluster are merged together. This step allows patients with similar causing genes to be analyzed together. After that, 0–1 centrality features extracted from the fused networks are used to train the logistic models that calculate the probability of each genes being disease-associated in each fused network. Finally, an ensemble strategy is performed by choosing the maximum probability obtained from different fused networks as the final probability of a gene being disease-associated.

In the experiments conducted on BC, TC and AD, our EdgCSN is much better than the competing algorithms in terms of AUC scores. Further analysis of the top 10 unknown genes also illustrate that EdgCSN is capable of predicting novel disease genes. Our study has provided insight into how clustering patient samples might improve the prediction of disease genes.

## Conclusions

Our EdgCSN use ensemble learning to predict disease genes from clustered sample-based networks. In the future, the strategies used for clustering can be further improved. For instance, Eq. (2) uses the expression data of all the genes to calculate the pairwise distances, and the results might be dominated by non-disease genes. We could reduce the number of genes used for clustering and choose those differentially expressed genes or marker genes that are associated with a specific subtype. These subsets of genes should improve the clustering results as well as the final prediction.

## Data Availability

The datasets generated and analyzed during the current study are available at: https://github.com/luoping1004/EdgCSN.

## Abbreviations

AD: Alzheimer’s disease
AUC: Area under the curve
BC: Breast cancer
DGN: Disease gene network
EdgCSN: **E**nsemble **d**isease **g**ene prediction by **C**linical **S**ample-based **N**etworks
FN: False negative
FP: False positive
FPR: False positive rate
LOOCV: Leave one out cross validation
PPI: Protein-protein interaction
ROC: Receiver operating characteristic
TC: Thyroid cancer
TN: True negative
TP: True positive
TPR: True positive rate
